# A novel posttranslational modification of histone, H3 S-sulfhydration, is down-regulated in asthenozoospermic sperm

**DOI:** 10.1007/s10815-021-02314-x

**Published:** 2021-10-18

**Authors:** Qi Qi, Hongjie Pan, Ning Jiang, Meixin Zhang, Shenfei Sun, Xiaofeng Wan, Fangxi Zhang, Lingling Zhang, Hua Diao, Jian Wang, Runsheng Li

**Affiliations:** 1 NHC Key Laboratory of Reproduction Regulation (Shanghai Institute of Planned Parenthood Research), Pharmacy School, Fudan University, 2140 Xietu Road, Shanghai, 200032 China; 2 NHC Key Laboratory of Reproduction Regulation (Shanghai Institute of Planned Parenthood Research), 2140 Xietu Road, Shanghai, 200032 China; 3 State Key Laboratory of Genetic Engineering, Institute of Biostatistics and Computational Biology, School of Life Sciences, Fudan University, Shanghai, 200438 China; 4 NHC Key Laboratory of Reproduction Regulation (Shanghai Institute of Planned Parenthood Research), School of Life Sciences, Fudan University, 2140 Xietu Road, Shanghai, 200032 China

**Keywords:** S-sulfhydrated proteome, H3S-sulfhydration, Asthenozoospermia, Spermatogenesis, H3.3

## Abstract

Oxidative stress is one of the major causes leading to male infertility including asthenozoospermia. Hydrogen sulfide (H_2_S) has been widely recognized to be a potent antioxidant whose role is partially implemented by protein S-sulfhydration. However, protein S-sulfhydration has not been reported in germ cells. Therefore, we investigated whether asthenozoospermia could be associated with sperm protein S-sulfhydration. S-sulfhydrated proteins in human sperm were enriched via biotin-switch assay and analyzed using LC-MS/MS spectrometry. Two hundred forty-four S-sulfhydrated proteins were identified. Importantly, we validated that sperm histones H3.1 and H3.3 were the S-sulfhydrated proteins. Their S-sulfhydrated amino acid residue was Cysteine111. Abundances of S-sulfhydrated H3 (sH3) and S-sulfhydrated H3.3 (sH3.3) were significantly down-regulated in asthenozoospermic sperm, compared with the fertile controls, and were significantly correlated with progressive motility. Retinoic acid (RA) up-regulated level of sH3.3 in primary round spermatids and the C18-4 cells (a mouse spermatogonial stem cell line). Overexpression of the mutant H3.3 (Cysteine111 was replaced with serine) affected expression of 759 genes and raised growth rate of C18-4 cells. For the first time, S-sulfhydration H3 and H3.3 were demonstrated in the present study. Our results highlight that aberrant S-sulfhydration of H3 is a new pathophysiological basis in male infertility.

**Supplementary Information:**

The online version contains supplementary material available at 10.1007/s10815-021-02314-x.

## **Introduction**

Mammalian spermatogenesis, a precisely regulated developmental process generating sperm, consists of three distinct phases. The first phase refers to the mitotic division of spermatogonia physiologically resulting in accumulation of germ cells depending on renewal and differentiation of spermatogonial stem cells (SSC). The second stage is meiosis, in which spermatocytes undergo two rounds of mitosis to produce haploid spermatids. The final one is spermiogenesis, wherein the round spermatids (rST), known as haploid spermatids, undergo a complex differentiation process to develop into spermatozoa, including chromatin remodeling, nuclear elongation, and flagellum development. A hallmark of mammalian sperm is the highly compact and condensed structure of chromatin, in which depending on the species, approximately 90–99 % of histones are replaced by protamines [1]. Distinct posttranslational modifications (PTMs) of histones in spermatogenesis are currently accepted to facilitate the chromatin remodeling and histone-to-protamine transition [1, 2]. Impaired spermatogenesis causes male infertility. Around 15% of couples at reproductive age present with infertility, and about half of the infertility are associated with male partner. Asthenozoospermia, a common male infertility, is characterized by both reduced sperm motility and normal concentrations of sperm (>15 million per matozoa/ml), and is defined as percentage of progressively motile (PR%) spermatozoa < 32% [3]. The molecular basis of asthenozoospermia is largely elusive.

Human spermatozoa are extremely vulnerable to oxidative attack because they contain little cytoplasm sequestering antioxidants. The oxidative stress (OS)–mediated damage to sperm has been considered as one of the leading causes for male infertility [4]. Defective mouse spermatogenesis can be caused by knockout of antioxidative genes, leading to an excessive production of reactive oxygen species (ROS) [5]. Asthenozoospermic sperm expressed some down-regulated antioxidative genes [6]. OS is accepted as the target for clinical treatment of asthenozoospermia [7].

Comparative proteomic analysis widely revealed altered expression of some proteins in asthenozoospermic sperm [8–10], indicating that sperm with poor motility might be caused by the lower expression of tubulin with structural defects in sperm flagellum. An altered expression of histone was detected in asthenozoospermic sperm by proteomic study [11]. Additionally, the aberrant expression of PTMs of proteins including phosphorylation [12], sumoylation [13], glutarylation [14], and hydroxyisobutyrylation [15] was associated with poor sperm motility. Interestingly, characterization of human sperm lysine acetylproteome revealed that protein acetylation was essential for sperm motility [16]. Study of the global protein phosphorylation landscape of spermiogenesis showed wide phosphoregulation across a diverse range of processes during spermiogenesis [17]. However, these proteomic studies did not show any specific protein whose function was actually regulated by these PTMs in germ cells. Additionally, the links between ROS and the PTMs of proteins have not been established in asthenozoospermic sperm.

Hydrogen sulfide (H_2_S) exerted a wide range of physiological and cytoprotective functions in the biological systems via its potent antioxidative capability [18]. The asthenozoospermic patients exhibited decreased concentration of H_2_S in their seminal plasma, while supplying exogenous H_2_S to semen improved sperm motility of the asthenozoospermic patients [19]. However, the mechanism remains unknown regarding how H_2_S exerts its roles in germ cells. Signaling by H_2_S has been widely found in eukaryotic cells via protein S-sulfhydration [20–22], a PTM on thiol group of cysteine residues that converts Cys-SH to Cys-SSH. About 10–25% of proteins extracted from liver are S-sulfhydrated in physiological conditions [20]. An accumulating number of proteins have been recently validated to be S-sulfhydrated proteins with the identified sulfhydrated cysteine residues, and their sulfhydration has wide and important functions including regulating redox balance [21, 23]. However, protein S-sulfhydration has not been reported in germ cells. We revealed S-sulfhydrated proteome consisting of 244 proteins of human sperm in the present study. They included most of ROS-associated human sperm reported elsewhere [24]. Importantly, for the first time, we demonstrated that histone H3 was an S-sulfhydrated protein in the present study. We further observed that the level of sH3 and sH3.3, a H3 variant, was positively correlated with percentage of sperm with progressive motility, respectively. Our findings highlighted a novel pathophysiological basis for asthenozoospermia.

## Experimental procedures

### Reagents, cells, and mice

Anti-H3.3antibody(#ab176840), anti-H3 antibody(#ab1971), anti-biotin antibody(#ab1227), anti-H3K4me3(#ab185637), and anti-H3K9me3(#ab8898) were purchased from Abcam (USA). GDNF was purchased from Peprotech (#450-44, USA). Male C57BL/6J mice were purchased from SIPPR-BK Animal Company (Shanghai, China).

The C18-4 cells was kindly provided by Prof. Zuping He, who was a principal investigator in Renji-Med X Clinical Stem Cell Research Center, Renji Hospital, School of Medicine, Shanghai Jiao Tong University. The C18-4 cells were grown as described by He et al. [25].

### Semen sample collection

This study (PJ2019-05) was approved by the Ethics Committee of Shanghai Institute of Planned Parenthood Research/World Health Organization (WHO) Collaborating Center on Human Research. Written informed constructs were obtained from the semen donors involved in the study. All the methods used in the present study were performed in accordance with the Declaration of Helsinki. The donors were recruited in compliance with the “WHO Laboratory Manual for the Examination and Processing of Human Semen” (Fifth edition). Semen samples were obtained by masturbation after 3–5 days of sexual abstinence. Semen samples which contained leukocytes were excluded from our study. Spermatozoa motility was assessed by the computer-assisted sperm assay (CASA) method according to World Health Organization guidelines, equipped with a camera (acA780-75gc, Basler, Germany), and a 20-fold objective, a camera adaptor (Eclipse E200, Nicon, Japan), operated by an SCA sperm class analyzer (MICROPTIC S.L.). Finally, the semen samples were collected from 26 normozoospermic men (24–45 years old, mean ± SEM: 31.54 ± 5.04 years old) and 24 asthenozoospermic patients (24–39 years old, mean ± SEM: 32.25 ± 4.78 years old) (Table S3), and used in the present study. The normozoospermic men had known reproductive histories in the past 2 years and progressive motility ≥32%, while the asthenozoospermic men had a progressive motility <32% (Table S3).

### Direct swim-up of spermatozoa

The direct swim-up of spermatozoa from semen was performed according to the “WHO Laboratory Manual” (5th edition). Briefly, place 2 ml of liquefied semen in a sterile 15-ml conical centrifuge tube, and gently layer 2 ml of modified HTF medium (#ART-1023, SAGE) with 5% human serum albumin solution (#10064, Vitrolife) over it. After sperm were incubated at 37 °C with 5% CO_2_ in humidified air for 1 h, gently collected the uppermost 1 ml of medium containing highly motile sperm cells and 1 ml semen at bottom of the tube, and used them as sperm with high motility and low motility, respectively. Centrifuged at 500*g* for 5 min and discarded the supernatant. The pellets were used for preparation of lysates.

### Biotin-switch assay

S-sulfhydrated proteins in human spermatozoa were detected using biotin-switch assay as described previously with minor modifications [20]. Briefly, 20 million spermatozoa were centrifuged at 2000 *g* for 5 min and supernatant removed, and spermatozoa were then resuspended in the lysis buffer (250 mM HEPES pH 7.7, 1 mM EDTA, 0.1 mM neocuproine, 1% Triton, 2.5% SDS) added with a cocktail of protease inhibitors (Sigma). The lysates were incubated for 5 min at room temperature and then centrifuged at 2000 *g* for 5 min. The supernatant was collected, and its protein concentration was adjusted to less than 0.5 mg/ml in each sample. Proteins were then precipitated using 4 volumes of ice-cold acetone for 20 min at −20°C, centrifuged at 2000 *g* for 5min at 4 °C, washed twice with 70% acetone, and dried out. The pellets were resuspended in HEN medium (250 mM HEPES pH 7.7, 1 mM EDTA, 0.1 mM neocuproine) containing 2.5% SDS. Free thiols of proteins were blocked with a rapidly thiol-reactive agent MMTS (20 mM) (#23011, Thermo Scientific, CHE) for 30 min at 50 °C. After the reaction, the proteins were precipitated with acetone as described above in order to remove excess MMTS, resuspended in HEN medium containing 1% SDS. Next, the proteins solution was added with 1mM biotin-HPDP (#A8008, APExBIO, USA) and incubated for 1 h at 25 °C to achieve biotinylation. The biotinylated proteins were separated by SDS-PAGE and finally detected with anti-biotin antibody or the indicated antibodies in Western blotting analysis.

### Cysteinyl labeling assay

We also detected S-sulfhydrated proteins in human spermatozoa using cysteinyl labeling assay as described elsewhere [26] with minor modifications. Briefly, 20 million spermatozoa were lysated in lysis buffer. The lysate was added with 2mM IAA for 1 h at room temperature. Cold acetone of double volume was then added into the sample. Then, the samples were precipitated at −20 °C for 20 min. After centrifuged at 12000 rpm at 4 °C for 10 min, the precipitation was diluted in HEN buffer with 1 mM DTT at room temperature for 30 min. A total of 3 mM biotinylated IAP was next added into the sample at room temperature for 1 h. Biotinylated proteins were enriched by using streptavidin-Sepharose beads for 16 h at 4 °C on a rotating wheel, with sequential rounds of centrifugation (12,000 *g*, 1 min, 4 °C) using PBS to wash the beads. The beads were resuspended in 20 μl of 4×Laemmli sample buffer and heated at 90 °C for 1 min. The biotinylated proteins were separated by SDS-PAGE and finally detected with anti-biotin antibody or the indicated antibodies in Western blotting analysis.

### Analysis of immunoprecipitation

Proteins from sperm or the C18-4 cells were biotinylated as described above. Protein concentration was adjusted to 0.1 mg/ml using HEN/10 media (10× dilution of HEN) containing 1% SDS. Three volumes of neutralization buffer (20 mM HEPES pH 7.7, 100 mM NaCl, 1 mM EDTA and 0.5% Triton X-100) were added. The mixture was separated into two parts. One part was added 2× SDS sample buffer for loading control, while another was incubated overnight at 4 °C with a specific antibody (1:1000) and 50 μl of protein A/G (#ab193262, Abcam, USA) per ml. Beads were previously washed twice with the neutralization buffer and centrifuged at 200 *g* for 10 s. Once the incubation terminated, the beads were washed 5 times with 500 μl of Wash buffer (the neutralization buffer containing 600 mM NaCl). Proteins were eluted with 1× SDS sample buffer containing 2 mM DTT. Samples were boiled for 5 min at 100 °C and centrifuged at 14000 *g* for 5 min. The supernatant was collected, separated by SDS-PAGE (10%), and detected via Western blotting analysis or silver staining. The gel with silver staining was excised, and applied for proteomic analyses which were performed as described below. For each of these experiments, 3 fertile ejaculates (from different donors) were pooled.

### Silver staining

After proteins were separated by SDS-PAGE, whole gel was washed with water for 5 min, and then soaked into blocking buffer (50% ethanol, 8% acetic acid, 0.4% formaldehyde) for 2 h. After washed with 35% ethanol for 3 times, the gel was soaked into staining buffer (10 mg/ml silver nitrate, 0.4% formaldehyde) for 30 min. After washed with water for 2 times, it was then soaked into cultivating buffer (0.12 g/ml sodium carbonate, 0.4% formaldehyde) until bands appeared. Finally, the gel was placed into termination buffer (50% ethanol, 8% acetic acid) for 5 min. The PAGE was scanned by Tanon scanner 5200.

### Identification of the sulfhydrated amino acid residue in H3.1 and H3.3

All the constructs that expressed wild-type and mutant H3.3 and H3 were purchased from Genomeditech (Shanghai, China). H3.1 was mutated in the three ways: C97 was replaced with serine (C97S), C111 was replaced by serine (C111S), and both of C111 and C97 were mutated to serine (double mutations). H3.3 was mutated at C111, which was also replaced with serine. All the expression constructs were generated based on pCMV2-FLAG tag (Promega, USA).

Transfection of the C18-4 cells with the H3 expression constructs via Lipofectamine 3000 (Invitrogen, Shanghai, China) was carried out according to the manufacturer’s protocol. Forty-eight hours later, the cells were harvested for preparation of lysate using the above lysis buffer. Wild-type and mutant H3.1 or H3.3 were immunoprecipitated using anti-FLAG antibody, and next subjected to biotin-switch assay. The biotinylated H3 was enriched via the IP using anti-biotin antibody, and finally detected using anti-FLAG antibody in Western blotting assay as described above.

### Primary germ cell preparation and treatment of rST with RA

SG cells were isolated from 8 days postpartum (dpp) mice [27]. The method of STA-PUT was used to isolate pacSC, rST, and eST. They were characterized as previously described [27, 28]. pacSC were from 17 dpp mice. rST and eST were from 56–70 dpp mice. After separated via gravity sedimentation, rST were pelleted via a centrifugation at 500 *g* for 5 min, cultured in DMEM (10% PBS) medium with 2 mM L-glutamine, 100 units/ml penicillin, and 100 mg/ml streptomycin at 37 °C with 5% CO_2_ in humidified air. Three hours later, RA (#r2625, Sigma-Aldrich, USA) diluted in ethanol was added to the culture medium to make a final concentration of 0.3 μM or 1 μM. Twenty-four hours later, the cells were harvested for measurement of sH3.3 expression.

### Treatment of testis with NaHS

Mice aged 4 weeks were sacrificed by cervical dislocation and then put them into 75% ethanol. The abdominopelvic cavity was opened using sterile scissors and forceps, and then the testis was pulled out. The testicular tunica albuginea was removed by puncturing the tissue and the loose seminiferous tubules were collected. The tubules were transferred to a new petri dish containing 2 ml of DMEM/F12 with gentamicin (0.02 g/l; Sigma-Aldrich). The seminiferous tubules were cut up, dispersed, and evenly distributed into 3 dishes in a humidified atmosphere at 34°C with 5% CO_2_. Three hours later, NaHS (50 or 100 mM; Sigma-Aldrich) was added to the medium. After 24-h treatment, the pieces of testis were collected for measurement of sH3.3 expression

### Infection of the C18-4 cells, cell number determination, qRT-PCR, and Western blotting assay

The recombinant Lentivirus that expressed the chimeric human H3.3 protein (wild-type or the mutant H3.3 with C111S) with a FLAG tag in the N-terminal of H3.3 was purchased from Kangchen Bio-tech (Shanghai, China). The ectopic expression of H3.3 and the mutant H3.3 by viral infection in the C18-4 cells were performed according to the manufacturer’s instructions. Cell number determination, qRT-PCR, and Western blotting assay were carried out as described previously [29].

### Mass spectrometry experimental design and statistical rationale

#### Sample preparation

Human sperm lysate was prepared from ten pooled sperm samples and separated by SDS-PAGE. Gel pieces were cut, destained for 20 min in 100 mM NH_4_HCO_3_ with 30% acetonitrile, and washed with Milli-Q water until the gels were fully destained. The spots were then lyophilized in a vacuum centrifuge. The in-gel proteins were reduced with dithiothreitol (10 mM DTT/100 mM NH_4_HCO_3_ ) for 30 min at 56 ° C, then alkylated with iodoacetamide (200 mM IAA/100 mM NH_4_HCO_3_) in the dark at room temperature for 30 min. Gel pieces were briefly rinsed with 100 mM NH_4_HCO3 and ACN, respectively. Gel pieces were digested overnight in 12.5 ng/μl trypsin in 25 mM NH_4_HCO_3_. The peptides were extracted three times with 60% ACN/0.1% TFA. The extracts were pooled and dried completely by a vacuum centrifuge.

#### LC-MS/MS

The peptide of each sample was desalted on C18 Cartridges (Empore™ SPE Cartridges, Sigma), then concentrated by vacuum centrifugation and reconstituted in 10 μl of 0.1% (v/v) formic acid. MS experiments were performed on a Q ExactiveHF mass spectrometer that was coupled to Easy nLC (Thermo Scientific). Peptide was first loaded onto a trap column (100 μm×20 mm, 5 μm, C18) with 0.1% formic acid, then separated by an analytical column (75 μm×100 mm, 3 μm, C18) with a binary gradient of buffer A (0.1% formic acid) and buffer B (84% acetonitrile and 0.1% formic acid) at a flow rate of 300 nl/min over 60 min. The gradient was set as the following: 5–8% buffer B from 0 to 2min, 8 to 23% buffer B from 2 to 42 min, 23 to 40% buffer B from 42 to 50 min, 40 to 100% buffer B from 50 to 52 min, 100% buffer B kept until 60 min. MS data were acquired using a data-dependent top20 method dynamically choosing the most abundant precursor ions from the survey scan (350–1800m/z) for HCD fragmentation. A lock mass of 445.120025 Da was used as internal standard for mass calibration. The full MS scans were acquired at a resolution of 60,000 at m/z 200, and 15,000 at m/z 200 for MS/MS scan. The maximum injection time was set to 50 ms for MS and 45 ms for MS/MS. Normalized collision energy was 27 and the isolation window was set to 1.5 Th. Dynamic exclusion duration was 30 s.

#### Database search

The MS data were analyzed using MaxQuant software version 1.5.8.3. MS data were searched against the UniProtKB Human database (157600 total entries, downloaded in July, 2017). The trypsin was selected as digestion enzyme. The maximal two missed cleavage sites and the mass tolerance of 4.5 ppm for precursor ions and 20 ppm for fragment ions were defined for database search. Carbamidomethylation of cysteines was defined as fixed modification, while acetylation of protein N-terminal and lysine and oxidation of methionine were set as variable modifications for database searching. The database search results were filtered and exported with <1% false discovery rate (FDR) at peptide level and protein level, respectively.

#### Protein structure modeling

The three-dimensional structure of H3.3 in nucleosome was generated using data from human nucleosome structure containing H3.3 (PDB ID: 5X7X) at 2.18 Å generated by PyMOL-1.5.0.3.

#### RNA sequencing and analysis

Total RNA was extracted from mutated and normal mice sample by RNeasyPlus Micro Kit (Qiagen, Wetzlar, Germany) following manufacturer’s instructions and reverse transcribed into cDNA libraries using the Ovation® RNA-Seq System V2 kit (NuGEN). Samples were sequenced with paried-ends reads (PE150) using IlluminaHiseq X-ten platform. The QC (quality control) analysis of the RNA sequencing data was performed using FastQC. The raw sequencing reads were pre-processed as follows: (1) removing adapter sequences, (2) removing reads with over 20 bp of low quality (Phred quality score < 20). The filtered clean reads were aligned to mouse reference genome (mm10) using Tophat2 and then the uniquely mapped reads were assigned to each annotated gene using featureCount. Statistical significant test of differentially expressed genes was performed by NOISeq with R [30]. Genes with absolute log2-transformed fold changes greater than 2 were regarded as differentially expressed genes and a threshold of *p* value < 0.05 was used. For significant DE genes, GO term pathway enrichment analysis was performed using the DAVID functional annotation tool.

## Results

### Analysis of sperm protein S-sulfhydration

**Fig. 1:**
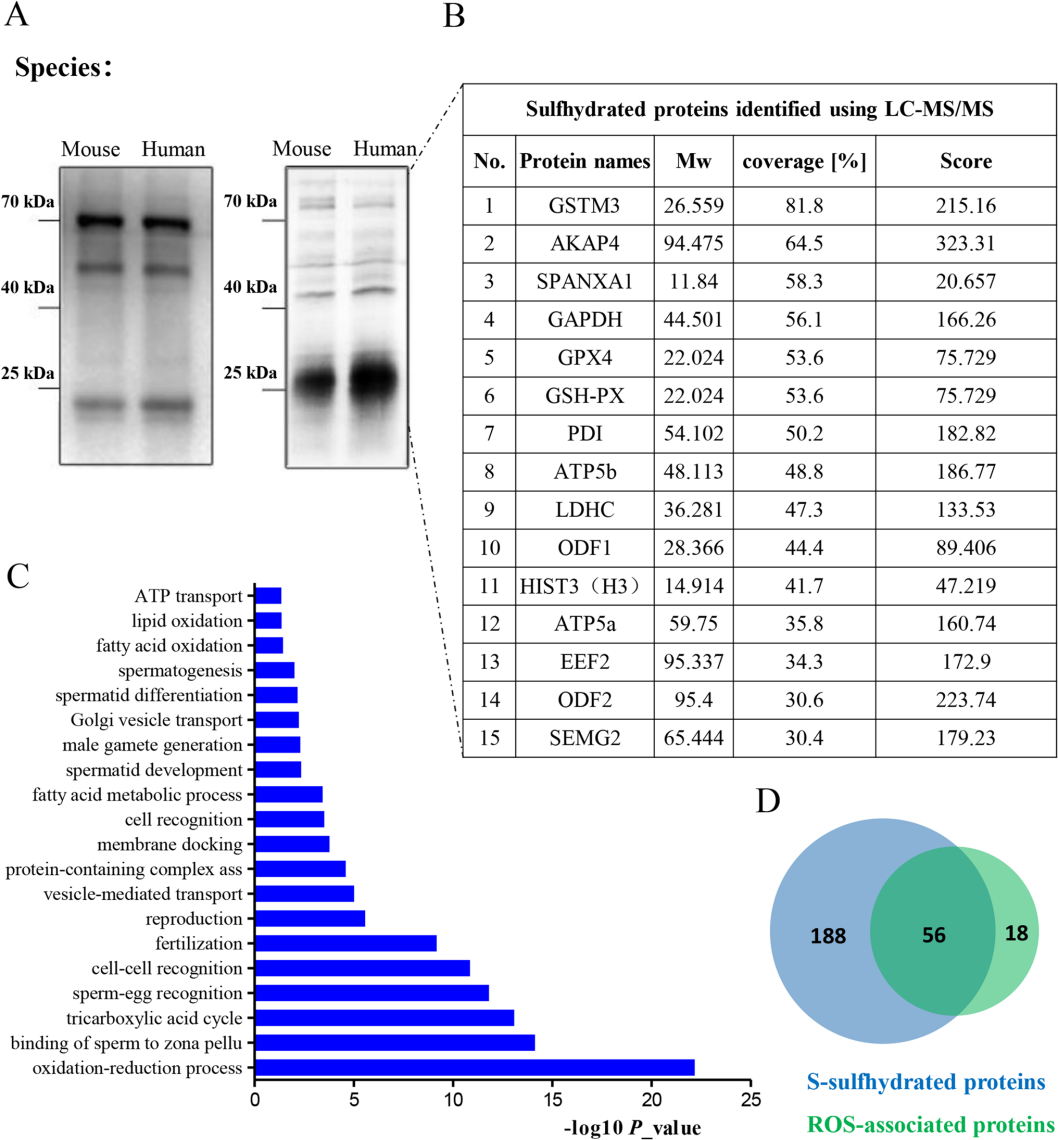
The S-sulfhydrated proteome of human sperm. (A) Detection of S-sulfhydrated proteins in human and mouse sperm. The lysates were prepared from pooled samples of human sperm (from ten fertile men) and mouse sperm (from three male mice), next subjected to the modified biotin-switch assay. The numerous sulfhydrated proteins were detected with anti-biotin antibody (left) or with silver staining (right). (B) LC-MS/MS of a subset of the S-sulfhydrated proteins in (A) identifies top notable S-sulfhydrated proteins, including GAPDH, GSTM3, and H3. (C) Biological processes enriched in the S-sulfhydrated proteins of human sperm. (D) Vann diagram depicting the relationship of sulfhydrated proteins and ROS-associated proteins of human sperm.

No specific antibody recognizing S-sulfhydrated proteins has been reported. Biotin-switch assay has been widely used for analysis of S-sulfhydrated proteins [20, 31, 32], in which free thiols (-SH) of proteins were blocked by a highly specific free sulfhydryl-reactive compound, methyl-methanethiosulfonate (MMTS), which did not interact with sulfhydrated thiols (-SSH) or any other forms of oxidized thiols (S-S, for example). The sulfhydrated thiols were then selectively labeled with N-(6-(biotinamido)hexyl)-3′-(2′-pyridyldithio)-propionamide (biotin-HPDP), a compound that interacts with sulfhydrated thiols in this assay, so that the sulfhydrated proteins were biotinylated. In order to examine protein S-sulfhydration in sperm, the extracts from mouse and human sperm were applied for the biotin-switch assay. The S-sulfhydrated proteins were enriched by immunoprecipitation (IP) using biotin antibody, separated via SDS–polyacrylamide gel electrophoresis (SDS-PAGE), and finally detected by anti-biotin antibody in Western blotting assay (Fig. 1A, left) and via silver staining (Fig. 1A, right), respectively. The results showed that sperm S-sulfhydrated proteins were detected both via Western blotting assay and silver staining.

To identify S-sulfhydrated proteins in human sperm, the S-sulfhydrated proteins stained with silver were subjected to in-gel trypsin digestion, next analyzed through liquid chromatography-tandem mass spectrometry (LC-MS/MS) followed by protein database searching of the acquired spectra. To control for nonspecific IP, IgG preimmune complex was also analyzed. The experiments were performed in three replicates, and each replicate was run through LC-MS/MS three times. Two hundred forty-four proteins were identified (Table S1) in the IP complex after (1) removing proteins that were not found in the replicate experiments and (2) subtracting common proteins that were found in the IgG preimmune complex.

In the list of proteins, S-sulfhydration of ATP synthase subunit alpha (ATP5a) [31, 33], glyceraldehyde-3-phosphate dehydrogenase (GAPDH) [31], and L-lactate dehydrogenase A chain (LDHA) [34] were elsewhere reported. In addition, we observed S-sulfhydration of Outer dense fiber protein 1 (ODF1) and ODF2, two proteins that stabilize the axoneme to maintain sperm motility [35]. Interestingly, we detected that sperm acrosin and acrosin-binding protein (ARCBP), which is needed for biogenesis of acrosome [36], was also S-sulfhydrated in sperm (Table S1), suggesting that the posttranslational modification is important for successful fertilization. Gene ontology (GO) analysis of the sulfhydrated proteome indicated that proteins were ontologically enriched for a series of functional clusters whose top three are oxidation-reduction process, binding of sperm to zonapellucida, and tricarboxylic acid cycle (Fig. 1C).

Expression of 74 sperm proteins was associated with a high level of ROS in seminal ejaculates [24]. We analyzed the relationship of the ROS-associated sperm proteome with our S-sulfhydrated sperm proteome. The results showed that 75.7% (56/74) of ROS-associated proteins were S-sulfhydrated proteins (Fig. 1D), implying that altered protein S-sulfhydration is the way for spermatozoa to respond to OS.

### Levels of sH3 and sH3.3 in asthenozoospermic spermatozoa

**Fig. 2:**
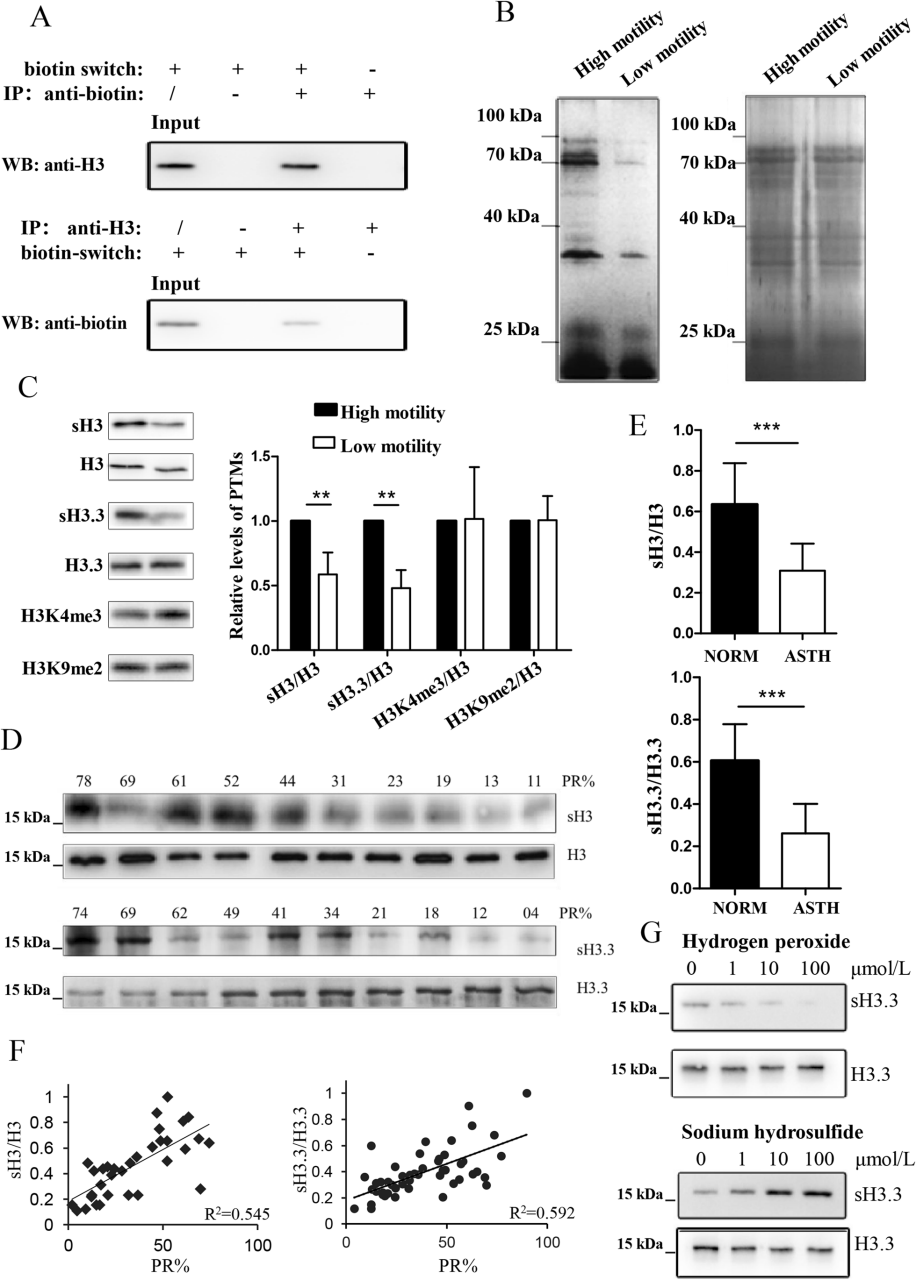
Association of sH3 and sH3.3 with asthenozoospermic sperm. (A) The validation of sH3.S-sulfhydrated proteins of human sperm lysates were biotinylated via biotin-switch assay, enriched via the IP based on anti-biotin antibody, and detected in Western blotting analysis using anti-H3 antibody (top panel). H3 in the lysate of pooled human sperm (*n*=5) was immunoprecipitated via anti-H3 antibody, and subjected to biotin-switch assay. The S-sulfhydrated H3 was detected in Western blotting analysis using anti-biotin antibody (bottom panel). (B) Detection of overall S-sulfhydrated proteins in sperm with different motility. Subpopulation of sperm with high and low motility from three fertile men was separated via swim-up assay, respectively, and their protein lysates were subjected to biotin-switch assay, detected with anti-biotin antibody in Western blotting assay (left panel). As a loading control, the proteins in the sperm lysates stained with Coomassie blue after separated via SDS-PAGE (right panel). (C) Expression of sH3 and sH3.3 in sperm with different motility. The biotinylated proteins in (B) were immunoprecipitated with biotin antibody, and further analyzed with anti-H3 and anti-H3.3 antibodies in Western blotting assay. In addition, the biotinylated protein in (B) from sperm with different motility was subjected in Western blotting assay using the indicated antibodies. The experiments were replicated in three fertile individuals. Error bar denotes mean ± SEM. *P < 0.05 and **P < 0.01. (D) H3 and H3.3 were enriched via IP with their antibodies from lysates of clinical sperm samples with different progressive motility (PR%), as indicated, next subjected to biotin-switch assay. One portion of the biotinylated proteins, as a loading control, were analyzed using anti-H3 and anti-H3.3 antibodies in Western blotting assay, while another was analyzed for detection of sH3 or sH3.3 using biotin antibody in Western blotting assay. (E) Relative expression of sH3 and sH3.3 in asthenozoospermic (ASTH) sperm (*N*=19 for sH3; *N*=24 for sH3.3) compared with the normozoospermic (NORM) controls (*N*=16 for sH3; *N*=26 for sH3.3). Error bar denotes mean ± SEM. ***P < 0.001. (F) Correlations among sperm sH3 (*n*=35), sH3.3 (*n*=50), and progressive motility were analyzed by linear regression. (G) Effects of H_2_O_2_ and H_2_Son expression of human sperm sH3.3. The cultured sperm was added with indicated concentration of H_2_O_2_ and NaHS, respectively, and 1 h later, subjected to analysis of sH3.3 expression. The analysis represented one of three independent experiments with almost the same results.

Spermatozoa histone H3 was identified as one of the S-sulfhydrated proteins (Fig. 1B). To further validate that H3 is an S-sulfhydrated protein, the crude sperm extract prepared from five pooled sperm samples from fertile men was applied for biotin-switch assay. The sulfhydrated proteins were immunoprecipitated using anti-biotin antibody. The IP was further analyzed with H3 antibody in Western blotting assay. The result showed that H3 was detected in the IP (Fig. 2A), indicating the presence of S-sulfhydrated H3 (sH3) in the extract. Additionally, the validation also started with enrichment of H3 via IP of the crude sperm extract using H3 antibody. The IP was next analyzed in biotin-switch assay, and applied for Western blotting assay using anti-biotin antibody. The result showed that H3 was recognized by anti-biotin antibody (Fig. 2A), indicating that sperm H3 is S-sulfhydrated.

The presence of S-sulfhydration of H3 was additionally evaluated by with cysteinyl labeling assay that utilizes a biotinylated iodoacetic acid (IAA) probe, which reacts through a nucleophilic substitution of the halide group by the H3-reactive thiol group, resulting in a stable thio-ether bond [26] (Fig S1A). We detected the presence of H3 after the lysate was applied for cysteinyl labeling assay in anti-H3 antibody-based immunoblotting analysis (Fig S1B). Similarly, H3 was detected by anti-biotin antibody in the WB assay, after the H3 was enriched from sperm lysate via IP, and next treated in cysteinyl labeling assay. Again, the results demonstrated that sperm H3 is a sulfhydrated protein.

The presence of S-sulfhydration of H3 was additionally evaluated by with cysteinyl labeling assay that utilizes a biotinylated iodoacetic acid (IAA) probe, which reacts through a nucleophilic substitution of the halide group by the H3-reactive thiol group, resulting in a stable thio-ether bond [26] (Fig S1A). We detected the presence of H3 after the lysate was applied for cysteinyl labeling assay in anti-H3 antibody-based immunoblotting analysis (Fig S1B). Similarly, H3 was detected by anti-biotin antibody in the WB assay, after the H3 was enriched from sperm lysate via IP, and next treated in cysteinyl labeling assay. Again, the results demonstrated that sperm H3 is a sulfhydrated protein.

We next studied the association of level of S-sulfhydrated protein with sperm motility. Normozoospermic sperm subpopulations with high motility and low motility were separated via the “swim-up” assay. The S-sulfhydrated proteins in their lysates were enriched using biotin antibody after biotin-switch assay, next detected with biotin antibody in Western blotting assay. As shown in Fig. 2B, a number of S-sulfhydrated proteins (around 15–100 KDa) were observed in the two sperm subpopulations. The overall level of S-sulfhydrated proteins was higher in sperm with high motility than in sperm with low motility.

Expressions of sH3 and S-sulfhydrated H3.3 (sH3.3) were measured in sperm with different motility. H3.3, a H3 variant played an important role in spermatogenesis [37], was thus selected in the analysis as well. H3 and H3.3 in sperm lysate were enriched via their antibodies-based IPs, respectively. The IPs were divided into two portions: one was applied for biotin-switch assay, and followed by an immunoblotting analysis using biotin antibody; the other was directly used as the loading control in Western blotting assay. Our results showed that abundances of sH3 and sH3.3 were significantly higher in sperm with high mobility than with low mobility (Fig. 2C). We also measured levels of other PTMs like H3K4me3 and H3K9me3 in the two subpopulations of normozoospermic sperm samples. However, no significant difference was detected. Together, these results indicated that expression of S-sulfhydration of proteins including sH3 and sH3.3 is positively associated with sperm motility.

Association of sH3 and sH3.3 with asthenozoospermia was next addressed. Levels of sH3 were measured in semen samples with different percentage of progressive motility (PR%) in Western blotting assay. Lower mean levels of sH3 and sH3.3 were found in asthenozoospermic men compared with fertile men (Fig. 2D). Statistical analysis showed that the mean levels of sH3 in patients (*n*=19) were 53.1% of that in the fertile controls (*n*=16). Additionally, mean levels of sH3.3 in patients (*n*=24) were only 42.3% of that in the fertile controls (*n*=26) (Fig. 2E). Correlation analysis results revealed that sperm levels of sH3 and sH3.3 correlated positively with progressive motility (Fig. 2F). Collectively, our study demonstrated that expression of sH3 and sH3.3 was down-regulated in asthenozoospermic sperm.

The effect of sperm redox status on levels of sH3 was next addressed via treating sperm with the oxidative agent hydrogen peroxide (H_2_O_2_) and NaHS, which has been widely used as a H_2_S donor in culture. H_2_S was shown to be an oxidants scavenger in sperm [19]. The results showed that H_2_O_2_ reduced the level of sH3 in a dose-dependent way (Fig. 2G). By contrast, NaHS raised the level of sH3 in a dose-dependent way. These results indicated that the level of sperm sH3 is under the control of redox status in a cellular context.

### Dynamics of sH3 and sH3.3 in spermatogenesis

**Fig. 3:**
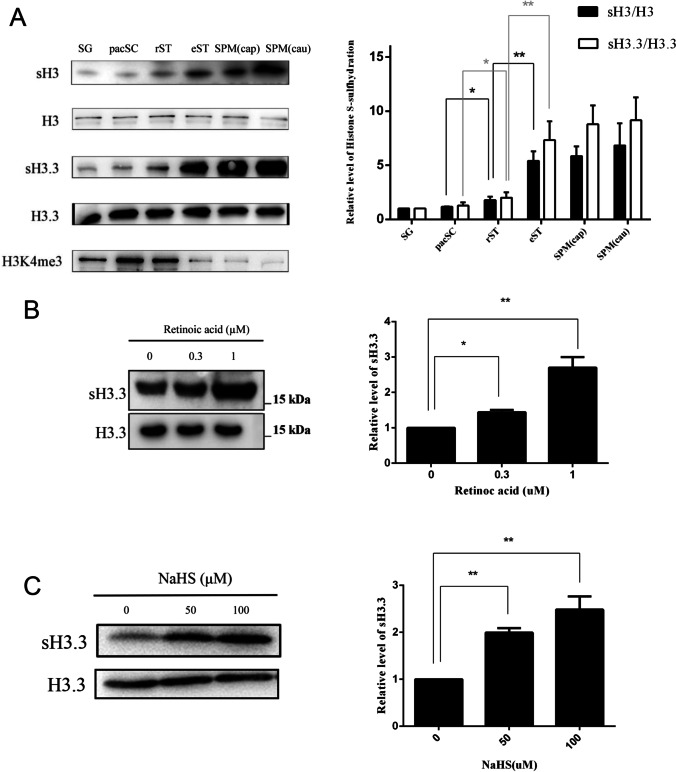
Analysis of expression of sH3 and sH3.3 in spermatogenesis and effect of RA and NaHS on level of sH3 in germ cells. (A) Spermatognia (SG), pachytene spermatocytes (pacSC), round spermatids (rST), and elongating/condensed spermatids (eST) were isolated and characterized as described in “Experimental procedures.” These germ cells, together with sperm from caput epididymis (SPM (cau)) and from cauda epididymis (SPM (cau)), were applied for preparation for protein lysates, subjected for measurement of expression of sH3 and sH3.3. (B) The incubated rST were treated with two indicated doses of RA for 24 h, then subjected for analysis of sH3.3 expression. (C) The incubated pieces of testis were treated with the indicated concentrations of NaHS for 24 h, and next subjected for measurement of expression of sH3.3. The experiments were repeated independently for three (for B and C) to four (for A) times. Error bar denotes mean ± SEM. *P < 0.05 and **P < 0.01.

We next approached the dynamic level of H3 S-sulfhydration during mouse spermatogenesis. Germ cells at different phases of spermatogenesis were first isolated, including spermatogonial cells (SG), pachytenespermatocytes (pacSC), which are at the prophase of the first meiotic division, round spermatids (rST), and elongating/condensed spermatids (eST). Both rST and eST are haploid germ cells. In addition, sperm from caput epididymis (SPM (cap)) and cauda epididymis (SPM (cau)) was isolated, respectively. Levels of sH3 and sH3.3 in the six types of germ cells were measured (Fig. 3A). These cells were applied for measurement of sH3 and sH3.3. Our results showed that all the types of germ cells expressed sH3 and sH3.3. A small but significant up-regulation of sH3 (by 151.2%) and sH3.3 (by 164.7%) was detected in rST, compared with pacSC. Notably, levels of sH3 and sH3.3 were significantly higher by 3.02 folds and 4.11 folds, respectively, in eST than in rST (Fig. 3A). As a control, expression of H3K4me3 was also measured and found to drop much in eST, compared to rST. No statistically significant difference was observed in expression of sH3, and sH3.3 was observed between eST and SPM (cap). Together, the results indicated spermiogenesis is the main stage for H3 and H3.3 to be S-sulfhydrated in spermatogenesis.

RA is a key physiological factor triggering differentiation of rST to eST [38]. We next investigated the effect of RA on H3.3S-sulfhydration in spermatids. The cultured rST were treated with RA soon after they were separated from testis. The results showed that 0.3μM and 1.0 μM RA raised expression of sH3.3 by 144.0% and 266.2% (Fig. 3B), respectively. Together, these results indicated that S-sulfhydration of H3.3 is induced by RA in rST.

We next studied whether H3.3 was susceptible to H_2_S-induced sulfhydration in a testicular context. The pieces of testis were incubated and treated with NaHS. The results showed that sH3.3 expression was raised significantly upon treatment with the H_2_S donor (Fig. 3C), suggesting that H3.3 S-sulfhydration is under the control of H_2_S signaling in spermatogenesis.

### Analysis of S-sulfhydrated amino acid residue in H3

**Fig. 4:**
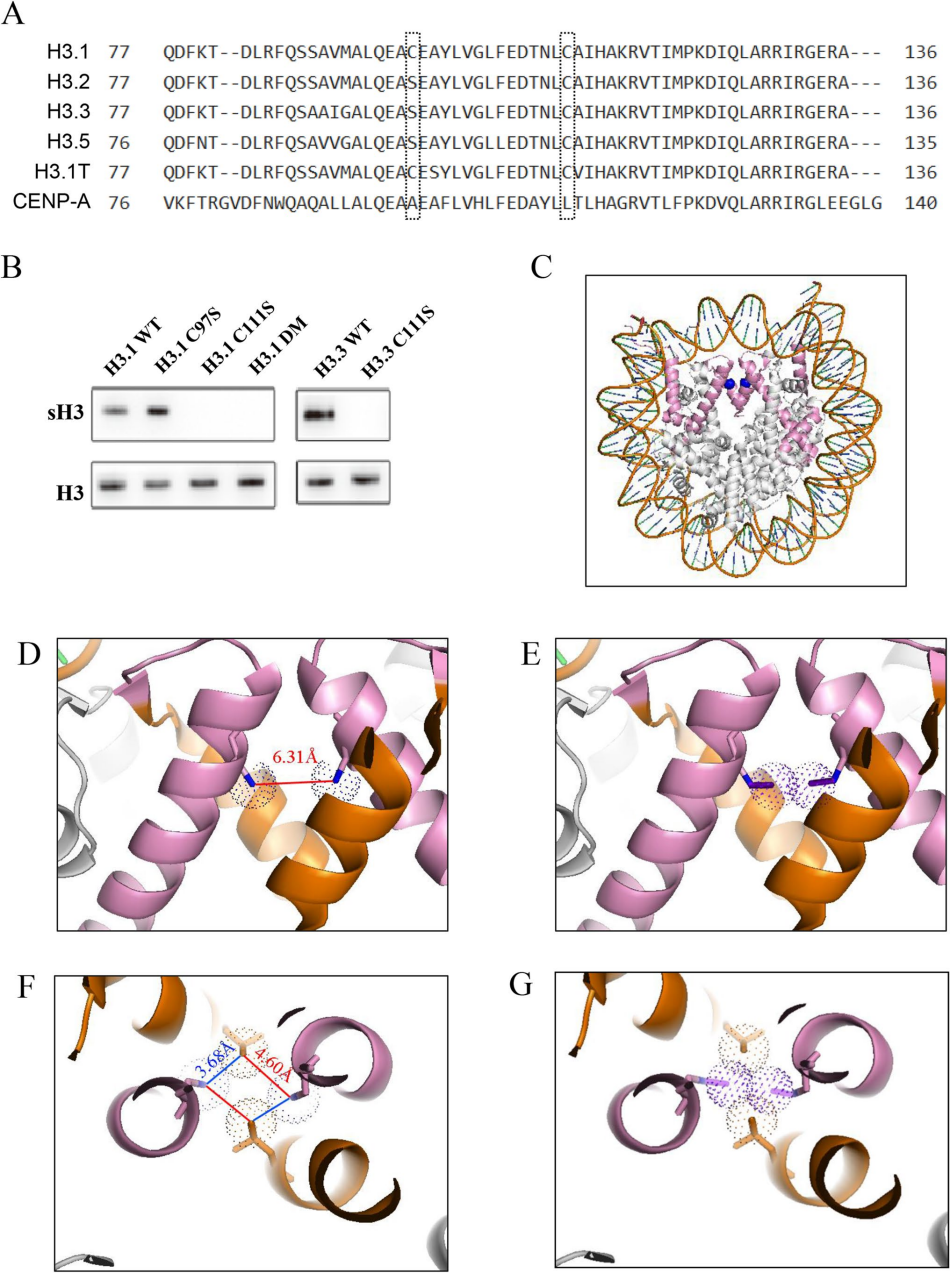
Analysis of effect of H3.3S-sulfhydration on structure of nucleosomal H3.3. (A) Sequence alignment of human H3 family. H3 family only contains two cysteine residues which were framed in the sequences. Note that C111 is conservative in five members of H3 family. (B) Effect of mutation of C97 and C111 on S-sulfhydration of H3.1 and H3.3. The C18-4 cells were transiently transfected with wild-type and mutant, as indicated, H3.1 and H3.3 expression constructs, respectively. Forty-eight hours later, the cells were harvested for measurement of levels of sulfhydrated wild and mutant H3.1 and H3.3. H3.1DM: the mutant H3.1 contains both C97S and C111S. (C) The structure of H3.3 in nucleosome, based on crystal structure solved at 2.8 Å [PDB ID:3AV2]. H3.3 (pink) forms as dimer in nucleosome, binding with H2A, H2B, and two H4. Cys111 (blue) locates in the center. (D) Range between two sulfur atoms of two C111. C111 locates in alpha helixes (85-114, pink), and is surrounded by alpha helix (120-132, orange). The distance between two sulfur atoms of two Cys111 is 6.31Å, as indicated. (E) Simulated structure of H3.3 when both Cys111 are S-sulfhydrated. The simulated electron cloud of outer sulfur atoms (red) is overlapped. (F) Distance between two sulfur atoms of two Cys111 and two nitro atoms of two R128. R128 locates in alpha helix (120-132, orange). The distance between each R128 and C111 is 3.68~4.60Å, showed by blue and red line. (G) The presence of overlapping electron cloud of the four atoms when both C111 are S-sulfhydrated.

At least 6 variants of H3 have been reported (Fig. 4A). The canonical H3.1 and H3.2 are expressed and deposited on nucleosomes during DNA replication [39]. The expression of H3.1t is testis-specific. Centromere protein A (CENP-A), a highly specialized variant, is only present at the centromere. Mammalian H3.3 is expressed throughout the cell cycle, and deposited by a DNA replication–independent nucleosome assembly pathway [39]. Only two cysteines, Cysteine 111 (C111) and Cysteine 97 (C97), are present in H3.

H3.1 has two cysteine residues. We studied which cysteine mutation could disrupt H3.1 sulfhydration in transient transfection assay (Fig. 4B). Serine was used to replace cysteine in the constructs expressing the mutant H3.1. Our results showed that the mutation of C111, but not C97, completely disrupted H3.1 sulfhydration. H3.3 has only one cysteine, C111. Similarly, H3.3 S-sulfhydration was fully abolished when its C111 was mutated. These results indicated that C111 is the site for S-sulfhydration both in H3.1 and H3.3.

We failed to directly detect S-sulfhydrated peptide digested from human sperm H3 via MS analysis. We first asked the question whether S-sulfhydration H3 could be directly observed by MS analysis. The key is whether the peptides containing the sulfhydrated C111 could be identified by MS after trypsin digestion. We next checked the *PeptideAtlas database (**http://www.peptideatlas.org/**)*, which is a multi-organism, publicly accessible compendium of peptides identified in a large set of tandem mass spectrometry proteomics experiments, for all possible peptides that could be identified by MS for H3 (https://db.systemsbiology.net/sbeams/cgi/PeptideAtlas/GetProtein?atlas_build_id=337&protein_name=P84243&action=QUERY). As shown in the above link and in Supplementary Fig. 2, the peptide between N-terminal 81 and 137 amino acid residues of H3 belongs to the category which is unlikely to be identified due to its length. C111 happens to fall into this peptide sequence, and S-sulfhydration of C111 thus could not be identified directly by MS.

In order to analyze the effect of S-sulfhydration of H3.3 on a nucleosomal structure, the structure of human H3.3-containing nucleosome was next stimulated based on its crystal structure solved at 2.8 Å [PDB ID: 3AV2] [40]. As shown in the nucleosome contains two H3.3 (Fig. 4C and D), the two C111 are located in the alpha helix (85-114) of H3.3, facing each other. The range between two sulfur atoms is 6.31 Å (Fig. 4D). Given an average distance of 2.04 Å between the two sulfur atoms which generates a disulfide bond [41], it is still too far for the two cysteines to form a disulfide bond. However, when both of C111 are S-sulfhydrated, the distance between the two outer sulfur atoms is narrowed to approximately 2.23 Å, which is probably close enough to form a disulfide bond (Fig. 4E). Therefore, our simulation analysis suggested that S-sulfhydration of H3.3 is beneficial to a formation of an inter-molecular disulfide bond between two nucleosomal H3.3 proteins.

On the other hand, our analysis of structure of two H3.3-containing nucleosome [40] also revealed another potential effect of S-sulfhydration of C111. The two alpha helixes (85-114) containing two C111 are close to another two helixes (120-132) of H3.3, which contains two Arginine128 (R128) (Fig. 4F). The region around C111 in these four helixes is important to form the H3-H3 hydrophobic four-helix bundle tetramer interface so as to hold together two histone H2A-H2B-H3-H4 tetramers [42]. Our analysis showed that the two nitrogen atoms of two R128 are very close to two sulfur atoms of two C111, and their distance is 3.68~4.60 Å (Fig. 4F). Most of N-H-S hydrogen bonds can form with a distance of 3.25~3.55 Å [43]. When both of C111 are S-sulfhydrated, the distance between two C111 and two R128 is much closer, so that the simulated electron cloud of 4 atoms overlaps each other (Fig. 4G). Therefore, these four residues can probably generate multiple inter-helical hydrogen bonds, thus stabilizing the structure of four-helix bundle tetramer, and the whole nucleosome.

### Effect of the C111 mutation of H3.3 on growth rate and gene expression of C18-4 cells

**Fig. 5:**
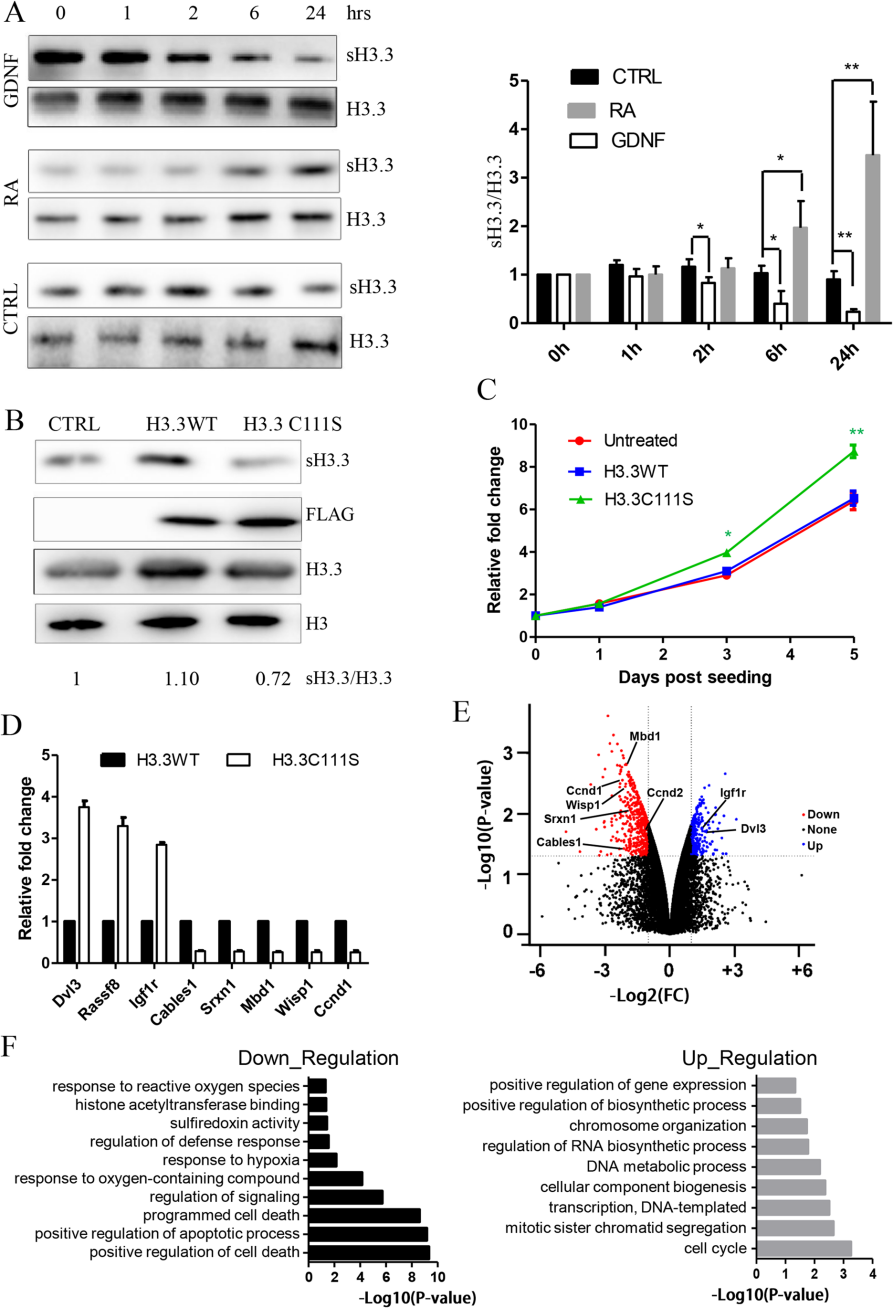
Effect of ectopic expression of the mutant H3.3 with C111S on cell growth rate and gene expression in the C18-4 cells. (A) Effects of GDNF and RA on sH3.3 expression in the C18-4 cells. Twenty-four hours after plating, the C18-4 cells were added with GDNF (50 ng/ml) and RA (1.0μM), respectively. The cells were harvested for measurement of sH3.3 expression at the indicated times after the treatment. Error bar denotes mean ± SEM. *P < 0.05 and **P < 0.01. (B) Expression of sH3.3 and H3.3 in the C18-4 cells infected with the recombinant Lentivirus that expressed wild-type and mutant (C111S) H3.3. The cultured C18-4 cells were harvested for the analysis when their confluence reached approximately 80–90%. These results represented one of three independent experiments with the similar data. (C) The growth rates of C18-4 cells. Untreated C18-4 cells and infected C18-4 cells which overexpressed wild-type or mutant H3.3 were seeded as described in “Experimental procedures,” and harvested for the counting of cell number at the indicated times after seeding. (D) Volcano plot showing differential expression of protein-coding genes between mutated and normal samples. Red and blue dots indicate significantly down-regulated (*p*<0.05 and log2FC<-1) and significantly up-regulated (*p*<0.05 and log2FC>1) differential expression, respectively. (E) RNA-seq data validation by quantitative RT-PCR. Expression of select up-regulated and down-regulated genes from the RNA-seq analysis was measured by quantitative RT-PCR in the C18-4 cells. (F) Biologic processes that are enriched in genes down-regulated (left) and up-regulated (right).

The presence of sH3.3 in spermatogonia (Fig. 3A) suggested that sH3.3 could play an important role in the phase of mitosis in spermatogenesis. We studied the hypothesis using SSC C18-4 cell line. Glial cell-line-derived neurotrophic factor (GDNF) is bona fide self-renewal factors of SSC, and promotes proliferation of C18-4 cells [25]. By contrast, RA signaling, which is a key physiological regulator of SSC differentiation, is also present in C18-4 cells [44]. The impact of GDNF and RA on sH3.3 expression was next investigated. GDNF was found to upgrade sH3.3 expression in a time-dependent way (Fig. 5A). A significant rise in level of sH3.3 was observed as early as 2 h after GDNF treatment. However, sH3.3 expression was reduced in the C18-4 cells when treated with RA in a time-dependent way. These results suggested that an altered sH3.3 expression is an important downstream event in signaling of GDNF and RA.

In order to explore the role of sH3.3 in the germ cells, we studied the effect of the H3.3 C111S mutant on growth rate of C18-4 cells. The C18-4 cells were thus infected with the recombinant Lentivirus that expressed wild-type H3.3 and a C111S-containing H3.3, respectively. No significant difference in the intensity of bands was detected with FLAG antibody between the two samples (Fig. 5B), indicating that the mutation did not affect H3.3 expression based on virus infection. Importantly, we detected that expression of sH3.3 was significantly higher in the C18-4 cells overexpressing wild-type H3.3 than those overexpressing the mutant H3.3 (Fig. 5B).

We next measured the cell number at different times after the infected and untreated C18-4 cells were seeded. We also did not detect any significant difference in cell number between C18-4 cells infected with wild-type H3.3-expressing virus and the untreated C18-4 cells (Fig. 5C). Strikingly, the mutant H3.3-expressing C18-4 cells grew faster than wild-type H3.3-expressing cells. The mutant H3.3-expressing cells were more than the controls, significantly by 25.8% and 36.1%, at the third and fifth day after the plating of cells (Fig. 5C). The results strongly suggested that sH3.3 is inhibitory to the growth of C18-4 cells, consistent with the repressive effect of GDNF on sH3.3 expression.

We next performed RNA sequencing analysis (RNA-seq) in order to study the mechanism underlying the promoting effect of C111S of H3.3 on the C18-4 cell growth rate. We found that expressions of 487 genes were down-regulated, while the other 272 genes were up-regulated (Fig. 5D, Table S2). Validation by quantitative RT-PCR was performed for some differentially expressed genes (DEGs), and our quantitative RT-PCR analysis confirmed the RNA-seq data (Fig. 5E).

Introduction of the mutated H3.3 reduced relative expression of Cyclin D1 (Ccnd1). Ccnd1 expression was inhibitory to growth of SSCs [45]. Rassf8 reduced the expression of ccnd1 when overexpressed in SSC [46], and its expression was also unregulated in the presence of the mutated H3.3 (Fig. 5E). Therefore, sH3.3 could regulate renewal of SSC via targeting the two genes. Some growth-inhibitory genes including Cdk5 and Abl enzyme substrate 1 (Cables1) [47] and ankyrin repeat domain 1 (Ankrd1) [48] were shown to be down-regulated in the presence of C111S (Fig. 5E). Among the list of unregulated genes, insulin-like growth factor 1 receptor (IGF-1R) is essential for the proliferation of mouse SSC by promoting the G2/M progression of the cell cycle [49]. Wnt1 inducible signaling pathway protein 1 (Wisp1) is required for proliferation of mesenchymal stem cells [50]. Dishevelled segment polarity protein 3 (Dvl3) repressed differentiation of mesenchymal stem cells inhibited via up-regulating Ccnd1 [51], and unregulated upon the ectopic expression of the mutant H3.3. Together, the mutation could promote the growth of C18-4 cells by regulating expression of these genes.

GO analysis on the down-regulated DEGs found many proteins involved in positive regulation of cell death, and positive regulation of apoptotic process that were highly expressed in C18-4 cells with overexpression of the mutant H3.3. Keeping in line with it, some significant terms associated with up-regulated mRNAs in the presence of the mutant H3.3 were cell cycle, mitotic sister chromatin segregation, and cellular macromolecule biosynthetic process.

## Discussion

We reported the human sperm S-sulfhydrated proteome including 244 proteins in the present study. GO analysis suggested that S-sulfhydrated proteins played important roles in spermatogenesis, spermiogenesis, and fertilization. S-sulfhydration of GAPDH significantly raised its enzyme activity [31]. Male mice with deficiency of GAPDH were infertile and had profound defects in sperm motility [52]. S-sulfhydration of ATP synthase [32] and LDHA [34] raised mitochondrial bioenergetics. Therefore, S-sulfhydration of these proteins is probably required for maintenance of optimal motility of sperm via regulating energy metabolism. Our study also revealed a new mechanism regarding why addition of exogenous H_2_S to semen improved the asthenozoospermic sperm motility [19]. Importantly, overall expression of sulfhydrated proteins including sH3/H3.3 is higher in sperm with high motility than with low motility (Fig. 2B–E), and levels of sH3 and sH3.3 are positively associated with sperm progressive motility (Fig. 2F). Our analysis also revealed that most of ROS-associated proteins are S-sulfhydrated proteins in human sperm (Fig. 1D). Expression of sH3.3 in male germ cells including sperm is unregulated by H_2_S, a potent antioxidant (Figs. 2G and 3C), while sperm sH3.3 was down-regulated by H_2_O_2_ (Fig. 2G), strongly suggesting that expression of sH3.3 is under control of redox status. H_2_S raised enzyme activities of ATP synthase [32] and LDHA [34] via up-regulation of their S-sulfhydration. Collectively, it is plausible that ROS represses H_2_S signaling, which in turn causes hypo-sulfhydration of proteins including H3/H3.3 in a subtype of asthenozoospermic sperm. Therefore, our study highlights that sH3.3/sH3 is potentially a novel biomarker for diagnosing etiology of asthenozoospermia.

To our knowledge, both protein S-sulfhydration in germ cells and H3/H3.3 S-sulfhydration have not been reported before. More than ten different PTMs of H3 were elsewhere reported [53, 54], Therefore, this work extends the catalogue of histone PTM sites in mammalian cells. Oxidative stress and ROS are emerging as important players, shaping the epigenetic landscape of the entire genome via different mechanisms including modification of H3 methylation and acetylation [55, 56]. Our study strongly suggests that sperm H3 S-sulfhydration is under the control of redox homeostasis, unravelling epigenetic mechanisms underlying the pathophysiology of male infertility. Some interesting questions have emerged from our study. For example, how do ROS-producing factors including smoking, alcohol, and inflammation affect sperm H3 S-sulfhydration? It is known that high levels of ROS can cause male infertility through not only by lipid peroxidation or DNA damage but also reduced total antioxidative capability in spermatozoa. What are their relationships with altered H3 S-sulfhydration? A few antioxidant medicines have been used to treat male infertility with different curative effects [57]. Can investigation of sperm sH3 before and after treatment allow a better understanding, monitoring, or selection of alternative antioxidant medicines? They are issues worth of investigation.

H3.3 has been reported to be important for spermatogenesis [37, 58, 59]. We reported that S-sulfhydration of H3 and H3.3 was detected throughout spermatogenesis in the present study. Noteworthy, RA, which is known to induce rST to differentiate into eST [38], also up-regulated their sH3.3 expression (Fig. 3B). Keeping in line with the observation, the level of sH3.3 was significantly higher in eST than in rST (Fig. 3A). Deficiency of mouse H3.3 resulted in an aberrant spermiogenesis including an impaired development of round spermatids and poor motility of sperm [58, 59]. Collectively, sH3.3 is probably required for RA-induced spermiogenesis. Distinct aberrant PTMs of histones in spermiogenesis resulted in infertile phenotypes including poor sperm motility, strongly suggesting they can affect sperm motility in the way depending on their roles in modulating gene transcription or disturbing sperm chromatin remodeling [60–64]. It is plausible that hypo-sulfhydration of H3.3 may cause asthenozoospermia in a similar way.

A hallmark of mammalian spermiogenesis is the stepwise completion of transition from histones to protamines in spermatids [1, 2]. During the process, vast majority of not only total histones but also levels of differentially modified histones were much reduced in eST, or mature sperm compared with rST [54, 62, 65–67]. Consistent with the reports, we also detected an expression of H3K9me3 in rST but it largely disappeared in eST (Fig. 3A). By contrast, the presence of increased abundance of sH3/sH3.3 in eST (Fig. 3A) strongly suggests that sH3/sH3.3 marks the retained nucleosomes. The retained nucleosomes have been revealed to distribute in genomic DNA in a well-organized manner [68, 69], implying the existence of machinery protecting retained histones from eviction. However, little is known currently regarding the mechanisms. Considering that histone acetylation per se attenuates the interplay between histone and DNA to facilitate histone removal [1], it is naturally tempting to speculate that the nucleosomes with an extra stabilizing mechanism can probably be exempted from histone removal. The most members of the mammalian H3 family contain one or two cysteine(s) in their protein core, and this feature is a hallmark property of H3, given all other histone proteins lack cysteine. Intriguingly, the mammalian H3 variants contain C111 that is located in their helix (85-114), the region where both H3 proteins are closely apposed in the nucleosome core particle [70]. The region immediately surrounding C111 is important to hold together two histone H2A-H2B-H3-H4 tetramers, because mutations of C111, for example, destabilized the H3-H3 hydrophobic four-helix bundle tetramer interface in vitro [71]. Therefore, Hake and Allis proposed that two C111 form an intermolecular disulfide bond within two H3 proteins in the same nucleosome, adding stability to the H3-H4 tetramer [42]. Our analysis showed the two C111 of nucleosomal H3.3 are 6.31 Åapart (Fig. 4D), basically excluding the possibility that they form the disulfide bond. However, once the two Cys111 are thio-modified, the distance is narrowed to 2.3 Å (Fig. 4E), thus probably generating an intra-nucleosomal disulfide bond. In addition, our analysis also suggested that S-sulfhydration of C111 is favorable to the formation of multiple inter-helical hydrogen bonds between them and R128 (Fig. 4G). The four-residue-based multiple hydrogen bonds have been reported to exist in the structures of the four-helix bundle tetramer [72, 73], causing the formation of a super-secondary structure of four-stranded coiled coil [74], and thus probably adds stability to the H3–H4 tetramer. Collectively, S-sulfhydration of C111 is likely to benefit the exemption of some nucleosomes from histone removal via strengthening nucleosomal stability.

sH3.3 expression in the C18-4 cells is oppositely regulated by GDNF and RA (Fig. 5A), two key factors for SSC self-renewal and differentiation. Our study is consistent with the hypothesis that sH3.3 is important for the control of fate determination of SSCs. Ectopic expression of mutant H3.3 with C111S unregulated growth rate of C18-4 cells, partially by modulating expression of positive regulation of cell death and positive regulation of apoptotic process–related genes and cell cycle–related genes (Fig. 5F). Therefore, sH3.3 is likely a suppressor for the mitotic division of differentiating spermatogonia. The role of sH3.3 in differentiation of SSC should be next addressed. Nevertheless, our study revealed a regulatory role of sH3.3 in transcriptome in the SSC line.

Genomic distribution of H3.3 is critical to its regulating role in gene transcription [37, 75], and is regulated by RA in the way depending on raising turnover of H3.3 in the differentiation of embryonic stem cells [76, 77]. A high turnover of H3.3 was detected in male meiosis [37] and spermiogenesis [69]. Considering that RA unregulated expression of sH3.3 in male germ cells found in the present study, whether/how S-sulfhydration of H3.3 affects genomic distribution of H3.3 should be next investigated in the different phases of spermatogenesis. C111 of H3.3 is located inside the protein, making it difficult to be accessible for interaction of modified nucleosomal C111 with any non-histone proteins. Therefore, H3.3 is likely to be sulfhydrated largely outside nucleosomes. Some other PTMs of H3 were finished also outside nucleosomes [78]. In this regard, one can envision that H3.3 S-sulfhydration selectively regulates the turnover rate and distribution of H3.3 probably via modulating interaction of H3.3 and its chaperone proteins that were detected in spermatogenesis [37, 79]. The hypothesis is worth a further approach.

In conclusion, for the first time, H3.3 and H3 are showed to be S-sulfhydrated proteins in the present study. We demonstrated that levels of sH3.3 and sH3 were down-regulated in asthenozoospermic sperm, suggesting that hypo-sulfhydration of H3 and H3.3 is a new biomarker for male infertility. sH3 has been detected in all the different mouse organs examined (data not shown). It is well known that oxidative stress is involved in initiation and progression of diabetes, neurodegenerative diseases, vascular disease, hypertension, aging, and many other pathologies. Therefore, it could be speculated that aberrant regulation of sH3 can shape epigenetic landscape, eventually making a significant contribution to the initiation and progression of distinct chronic diseases.

## Supplementary information

Supplementary Figure 1 Detecting of sperm sH3 via cysteinyl labeling assay. (A) Schematic representation of cysteinyl labeling assay. Free thiol and S-sulfhydrated thiol of proteins in cell lysate were acetylated by IAA. Acetylated S-sulfhydrated thiol is selectively reduced by DTT in order to be next labeled with biotin by IAP. Biotin-linked proteins were then immunoprecipitated and detected in Western blot. (B) S-sulfhydrated proteins of human sperm lysate were also biotinylated via cysteinyl labeling, enriched via the IP based on anti-biotin antibody, and detected in WB analysis using anti-H3 antibody (Top panel). H3 in human sperm lysate was immunoprecipitated via anti-H3 antibody, and subjected to cysteinyl labeling assay. The sH3 was detected in Western blotting analysis using anti-biotin antibody (Bottom panel). The results represented one of three independent experiments with the same conclusion. (PNG 358 kb)

High resolution image (TIF 15261 kb)

Supplementary Figure 2 Summary of five categories of the peptides from H3 analyzed via MS analysis**.** A schematic map from the *PeptideAtlas database (*http://www.peptideatlas.org/*)*, which is a multi-organism, publicly accessible compendium of peptides identified in a large set of tandem mass spectrometry proteomics experiments, for all possible peptides that could be identified by MS for H3. The amino sequence positions are shown on the top, and the tryptic peptides are shown as colored blocks in the bottom along the sequence position. Different colors indicate different possibility of being identified by MS. There were five categories of the peptides: A, Observed peptide with single genome mapping; B, Signal peptide annotated in SwissProt; C, Protein coverage by observed peptides; D, Unlikely due to SSR; E, Unlikely due to Length. The peptide sequence between the amino acid residues 81 to 137 belongs to the category E, which is unlikely to be identified due to its length. (PNG 88 kb)

High resolution image (TIF 10281 kb)

ESM 1 (XLSX 30 kb)

ESM 2 (XLSX 78 kb)

ESM 3 (XLSX 12 kb)

## Data Availability

The data used to support the findings of this study are available from the corresponding author upon request.

## Abbreviations

ACRBP: Acrosin binding protein
ANKRD1: Ankyrin repeat domain 1
ASTH: Asthenozoospermic
ATP5A: ATP synthase F1 subunit alpha
Biotin-HPDP: N-(6-(biotinamido)hexyl)-3′-(2′-pyridyldithio)-propionamide
CABLES1: Cdk5 and Abl enzyme substrate 1
CCND: Cyclin D
CENP-A: Centromere protein A
dpp: Days postpartum
DEG: Differentially expressed genes
DVL3: Dishevelled segment polarity protein 3
eST: elongating/condensed spermatids
GAPDH: Glyceraldehyde-3-phosphate dehydrogenase
GDNF: Glial cell–derived neurotrophic factor
GO: Gene ontology
H_2_O_2_: Peroxide
H_2_S: Hydrogen sulfide
H3: Histone3
HirA1: Hira domain-containing protein
IAA: Iodoacetamide
IAP: Iodoacetyl-polyethylene glycol
IGF-1R: Insulin-like growth factor 1 receptor
IgG: Immunoglobulin G
IP: Immunoprecipitation
LC-MS/MS: Liquid chromatography-tandem mass spectrometry
LDHA: Lactate dehydrogenase A
MMTS: Methyl-methanethiosulfonate
NORM: Normozoospermic
ODF1/2: Outer dense fiber protein 1/2
OS: Oxidative stress
PTM: Posttranslational modifications
RA: Retinoic acid
rST: Round spermatids
RNA-seq: RNA sequencing
ROS: Reactive oxygen species
SDS-PAGE: SDS–polyacrylamide gel electrophoresis
sH3.3: S-sulfhydrated H3.3
sH3: S-sulfhydrated H3
SSC: Spermatogonial stem cells
Wisp1: WNT1 inducible signaling pathway protein 1

## References

[1] Bao J, Bedford MT (2016). Epigenetic regulation of the histone-to-protamine transition during spermiogenesis. Reproduction.

[2] Wang T (2019). Essential role of histone replacement and modifications in male fertility. Front Genet.

[3] Lu WH, Gu YQ (2010). Insights into semen analysis: a Chinese perspective on the fifth edition of the WHO laboratory manual for the examination and processing of human semen. Asian J Androl.

[4] Agarwal A, Gupta S, Sikka S (2006). The role of free radicals and antioxidants in reproduction. Curr Opin Obstet Gynecol.

[5] Smith TB (2013). Functional deletion of Txndc2 and Txndc3 increases the susceptibility of spermatozoa to age-related oxidative stress. Free Radic Biol Med.

[6] Chen K (2012). Low NRF2 mRNA expression in spermatozoa from men with low sperm motility. Tohoku J Exp Med.

[7] Meybodi AM (2012). Importance of sperm gluthatione treatment in ART. J Assist Reprod Genet.

[8] Cao X (2018). Proteomic profile of human spermatozoa in healthy and asthenozoospermic individuals. Reprod Biol Endocrinol.

[9] Macleod G, Varmuza S (2013). The application of proteomic approaches to the study of mammalian spermatogenesis and sperm function. FEBS J.

[10] Saraswat M (2017). Human spermatozoa quantitative proteomic signature classifies normo- and asthenozoospermia. Mol Cell Proteomics.

[11] Martinez-Heredia J (2008). Identification of proteomic differences in asthenozoospermic sperm samples. Hum Reprod.

[12] Parte PP (2012). Sperm phosphoproteome profiling by ultra performance liquid chromatography followed by data independent analysis (LC-MS(E)) reveals altered proteomic signatures in asthenozoospermia. J Proteome.

[13] Marchiani S (2011). Sumo1-ylation of human spermatozoa and its relationship with semen quality. Int J Androl.

[14] Cheng YM (2019). Lysine glutarylation in human sperm is associated with progressive motility. Hum Reprod.

[15] Cheng YM (2020). Posttranslational lysine 2-hydroxyisobutyrylation of human sperm tail proteins affects motility. Hum Reprod.

[16] Sun G (2014). Insights into the lysine acetylproteome of human sperm. J Proteome.

[17] Li Y (2019). The protein phosphorylation landscape of mouse spermatids during spermiogenesis. Proteomics.

[18] Xie ZZ, Liu Y, Bian JS (2016). Hydrogen sulfide and cellular redox homeostasis. Oxidative Med Cell Longev.

[19] Wang J (2018). Hydrogen sulfide as a potential target in preventing spermatogenic failure and testicular dysfunction. Antioxid Redox Signal.

[20] Mustafa AK (2009). H2S signals through protein S-sulfhydration. Sci Signal.

[21] Zhang D (2017). H2S-induced sulfhydration: biological function and detection methodology. Front Pharmacol.

[22] Ju Y et al. H(2)S-mediated protein S-sulfhydration: a prediction for its formation and regulation. Molecules. 2017 22(8) 10.3390/molecules22081334 PMC6152389 28800080

[23] Yang G (2013). Hydrogen sulfide protects against cellular senescence via S-sulfhydration of Keap1 and activation of Nrf2. Antioxid Redox Signal.

[24] Sharma R (2013). Proteomic analysis of human spermatozoa proteins with oxidative stress. Reprod Biol Endocrinol.

[25] He Z (2008). Gdnf upregulates c-Fos transcription via the Ras/Erk1/2 pathway to promote mouse spermatogonial stem cell proliferation. Stem Cells.

[26] Krishnan N (2011). H2S-Induced sulfhydration of the phosphatase PTP1B and its role in the endoplasmic reticulum stress response. Sci Signal.

[27] Pan H (2017). Ndrg3 gene regulates DSB repair during meiosis through modulation the ERK signal pathway in the male germ cells. Sci Rep.

[28] Buchou T (2017). Purification and analysis of male germ cells from adult mouse testis. Methods Mol Biol.

[29] Wang W (2009). NDRG3 is an androgen regulated and prostate enriched gene that promotes in vitro and in vivo prostate cancer cell growth. Int J Cancer.

[30] Tarazona S (2015). Data quality aware analysis of differential expression in RNA-seq with NOISeq R/Bioc package. Nucleic Acids Res.

[31] Jarosz AP (2015). Glyceraldehyde 3-phosphate dehydrogenase (GAPDH) is inactivated by S-sulfuration in vitro. Free Radic Biol Med.

[32] Modis K (2016). S-Sulfhydration of ATP synthase by hydrogen sulfide stimulates mitochondrial bioenergetics. Pharmacol Res.

[33] Paul BD, Snyder SH (2012). H(2)S signalling through protein sulfhydration and beyond. Nat Rev Mol Cell Biol.

[34] Untereiner AA (2017). H2S-induced S-sulfhydration of lactate dehydrogenase a (LDHA) stimulates cellular bioenergetics in HCT116 colon cancer cells. Biochem Pharmacol.

[35] Zhao W (2018). Outer dense fibers stabilize the axoneme to maintain sperm motility. J Cell Mol Med.

[36] Kanemori Y (2016). Biogenesis of sperm acrosome is regulated by pre-mRNA alternative splicing of Acrbp in the mouse. Proc Natl Acad Sci U S A.

[37] van der Heijden GW (2007). Chromosome-wide nucleosome replacement and H3.3 incorporation during mammalian meiotic sex chromosome inactivation. Nat Genet.

[38] Endo T (2017). Periodic production of retinoic acid by meiotic and somatic cells coordinates four transitions in mouse spermatogenesis. Proc Natl Acad Sci U S A.

[39] Henikoff S, Ahmad K (2005). Assembly of variant histones into chromatin. Annu Rev Cell Dev Biol.

[40] Tachiwana H (2011). Structures of human nucleosomes containing major histone H3 variants. Acta Crystallogr D Biol Crystallogr.

[41] Craig DB, Dombkowski AA (2013). Disulfide by Design 2.0: a web-based tool for disulfide engineering in proteins. BMC Bioinformatics.

[42] Hake SB, Allis CD (2006). Histone H3 variants and their potential role in indexing mammalian genomes: the “H3 barcode hypothesis”. Proc Natl Acad Sci U S A.

[43] Adman E, Watenpaugh KD, Jensen LH (1975). NH---S hydrogen bonds in Peptococcus aerogenes ferredoxin, Clostridium pasteurianum rubredoxin, and Chromatium high potential iron protein. Proc Natl Acad Sci U S A.

[44] Koli S, Mukherjee A, Reddy KVR (2017). Retinoic acid triggers c-kit gene expression in spermatogonial stem cells through an enhanceosome constituted between transcription factor binding sites for retinoic acid response element (RARE), spleen focus forming virus proviral integration oncogene (SPFI1) (PU.1) and E26 transformation-specific (ETS). Reprod Fertil Dev.

[45] He Z (2013). MiRNA-20 and mirna-106a regulate spermatogonial stem cell renewal at the post-transcriptional level via targeting STAT3 and Ccnd1. Stem Cells.

[46] Wang Y (2019). MicroRNA-322 regulates self-renewal of mouse spermatogonial stem cells through Rassf8. Int J Biol Sci.

[47] Arnason T (2013). Cables1 is a tumor suppressor gene that regulates intestinal tumor progression in Apc(Min) mice. Cancer Biol Ther.

[48] Jimenez AP (2017). The tumor suppressor RASSF1A induces the YAP1 target gene ANKRD1 that is epigenetically inactivated in human cancers and inhibits tumor growth. Oncotarget.

[49] Wang S (2015). IGF-1R signaling is essential for the proliferation of cultured mouse spermatogonial stem cells by promoting the G2/M progression of the cell cycle. Stem Cells Dev.

[50] Cernea M (2016). Wisp1 mediates Bmp3-stimulated mesenchymal stem cell proliferation. J Mol Endocrinol.

[51] He H (2015). miR-204-5p promotes the adipogenic differentiation of human adipose-derived mesenchymal stem cells by modulating DVL3 expression and suppressing Wnt/beta-catenin signaling. Int J Mol Med.

[52] Miki K (2004). Glyceraldehyde 3-phosphate dehydrogenase-S, a sperm-specific glycolytic enzyme, is required for sperm motility and male fertility. Proc Natl Acad Sci U S A.

[53] Sabari BR (2017). Metabolic regulation of gene expression through histone acylations. Nat Rev Mol Cell Biol.

[54] Tan M (2011). Identification of 67 histone marks and histone lysine crotonylation as a new type of histone modification. Cell.

[55] Thomas K (2017). The epigenetic landscape related to reactive oxygen species formation in the cardiovascular system. Br J Pharmacol.

[56] Sarah K (2016). Oxidative stress signaling to chromatin in health and disease. Epigenomics..

[57] Barati E (2020). Oxidative stress and male infertility: current knowledge of pathophysiology and role of antioxidant therapy in disease management. Cell Mol Life Sci.

[58] Yuen BT (2014). Histone H3.3 regulates dynamic chromatin states during spermatogenesis. Development.

[59] Tang MC (2015). Contribution of the two genes encoding histone variant h3.3 to viability and fertility in mice. PLoS Genet.

[60] Stiavnicka M (2020). H3K4me2 accompanies chromatin immaturity in human spermatozoa: an epigenetic marker for sperm quality assessment. Syst Biol Reprod Med.

[61] Yap DB (2011). Mll5 is required for normal spermatogenesis. PLoS One.

[62] Lu LY (2010). RNF8-dependent histone modifications regulate nucleosome removal during spermatogenesis. Dev Cell.

[63] Liu S (2017). Chromodomain protein CDYL acts as a crotonyl-CoA hydratase to regulate histone crotonylation and spermatogenesis. Mol Cell.

[64] Yu YE (2000). Abnormal spermatogenesis and reduced fertility in transition nuclear protein 1-deficient mice. Proc Natl Acad Sci U S A.

[65] Luense LJ (2016). Comprehensive analysis of histone post-translational modifications in mouse and human male germ cells. Epigenetics Chromatin.

[66] Dottermusch-Heidel C (2014). H3K79 methylation: a new conserved mark that accompanies H4 hyperacetylation prior to histone-to-protamine transition in Drosophila and rat. Biol Open.

[67] Song N (2011). Immunohistochemical analysis of histone H3 modifications in germ cells during mouse spermatogenesis. Acta Histochem Cytochem.

[68] Hammoud SS (2009). Distinctive chromatin in human sperm packages genes for embryo development. Nature.

[69] Erkek S (2013). Molecular determinants of nucleosome retention at CpG-rich sequences in mouse spermatozoa. Nat Struct Mol Biol.

[70] Luger K (1997). Crystal structure of the nucleosome core particle at 2.8 A resolution. Nature.

[71] Banks DD, Gloss LM (2004). Folding mechanism of the (H3-H4)2 histone tetramer of the core nucleosome. Protein Sci.

[72] Haworth NL (2019). Cross-strand disulfides in the hydrogen bonding site of antiparallel beta-sheet (aCSDhs): forbidden disulfides that are highly strained, easily broken. Protein Sci.

[73] Liu J (2006). A seven-helix coiled coil. Proc Natl Acad Sci U S A.

[74] Harbury PB (1993). A switch between two-, three-, and four-stranded coiled coils in GCN4 leucine zipper mutants. Science.

[75] Szenker E, Ray-Gallet D, Almouzni G (2011). The double face of the histone variant H3.3. Cell Res.

[76] Schlesinger S (2017). A hyperdynamic H3.3 nucleosome marks promoter regions in pluripotent embryonic stem cells. Nucleic Acids Res.

[77] Chen P (2013). H3.3 actively marks enhancers and primes gene transcription via opening higher-ordered chromatin. Genes Dev.

[78] Loyola A (2006). PTMs on H3 variants before chromatin assembly potentiate their final epigenetic state. Mol Cell.

[79] Rogers RS (2004). SUMO modified proteins localize to the XY body of pachytene spermatocytes. Chromosoma.

[80] Schon SB (2019). Histone modification signatures in human sperm distinguish clinical abnormalities. J Assist Reprod Genet.

