# Supplementary figures and images for: A novel posttranslational modification of histone, H3 S-sulfhydration, is down-regulated in asthenozoospermic sperm
Source: J Assist Reprod Genet. 2021 Oct 18;38(12):3175–93. doi: 10.1007/s10815-021-02314-x (PMC8666411; doi:10.1007/s10815-021-02314-x)

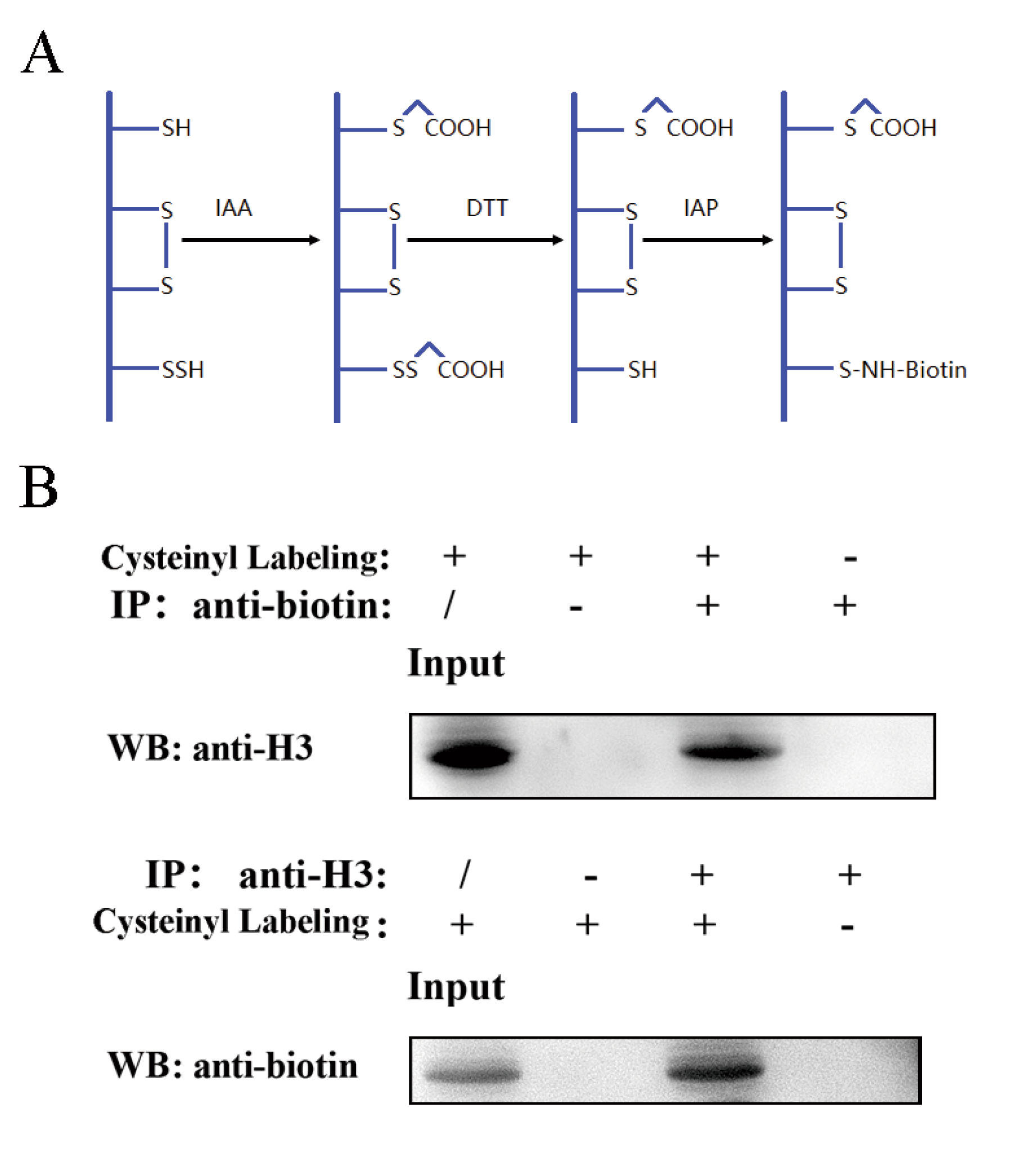

Supplement: Supplementary file 1 — Detecting of sperm sH3 via cysteinyl labeling assay. (A) Schematic representation of cysteinyl labeling assay. Free thiol and S-sulfhydrated thiol of proteins in cell lysate were acetylated by IAA. Acetylated S-sulfhydrated thiol is selectively reduced by DTT in order to be next labeled with biotin by IAP. Biotin-linked proteins were then immunoprecipitated and detected in Western blot. (B) S-sulfhydrated proteins of human sperm lysate were also biotinylated via cysteinyl labeling, enriched via the IP based on anti-biotin antibody, and detected in WB analysis using anti-H3 antibody (Top panel). H3 in human sperm lysate was immunoprecipitated via anti-H3 antibody, and subjected to cysteinyl labeling assay. The sH3 was detected in Western blotting analysis using anti-biotin antibody (Bottom panel). The results represented one of three independent experiments with the same conclusion. (PNG 358 kb) [file 10815_2021_2314_Fig6_ESM.png]

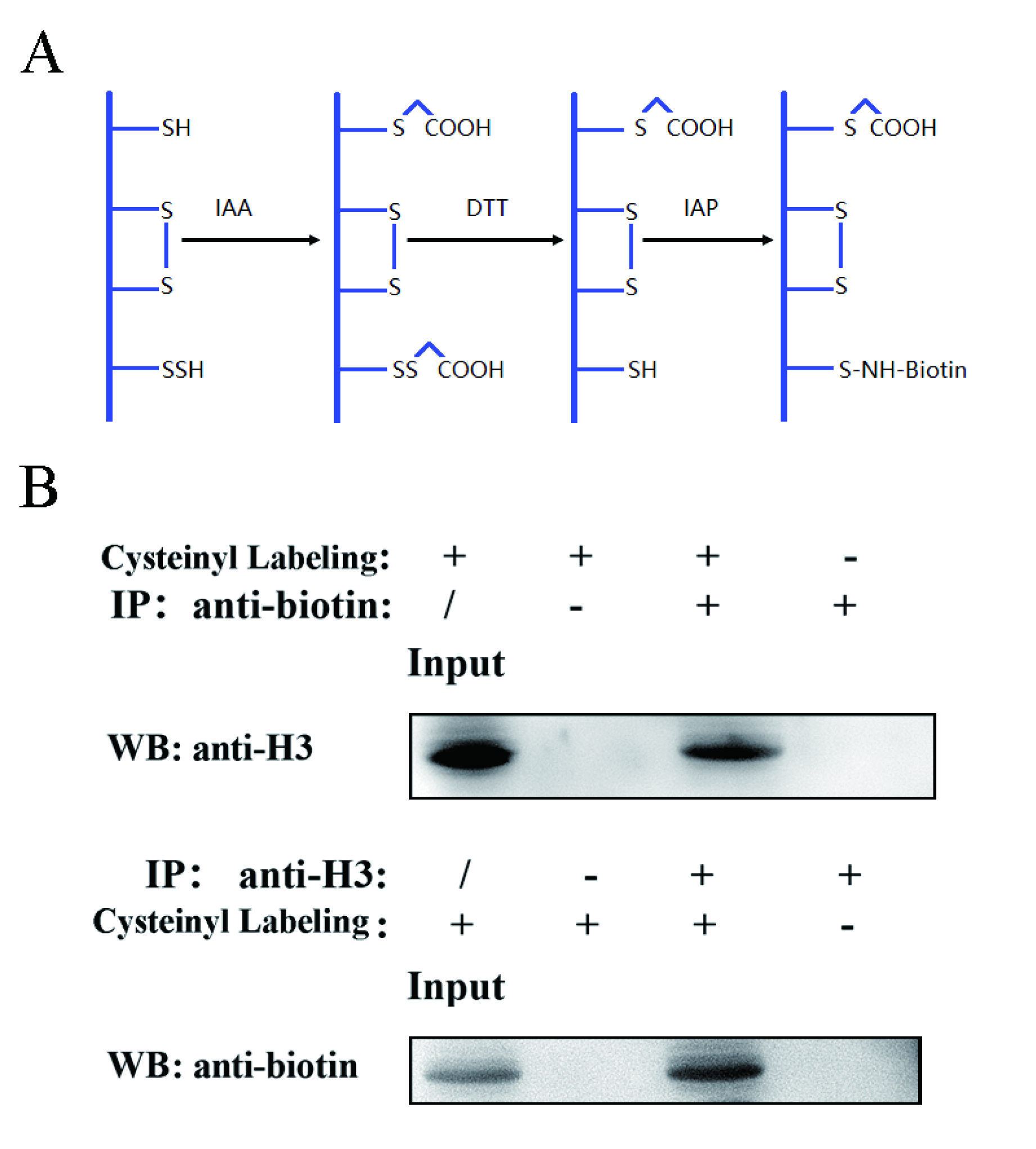

Supplement: Supplementary file 2 — High resolution image (TIF 15261 kb) [file 10815_2021_2314_MOESM1_ESM.tif]

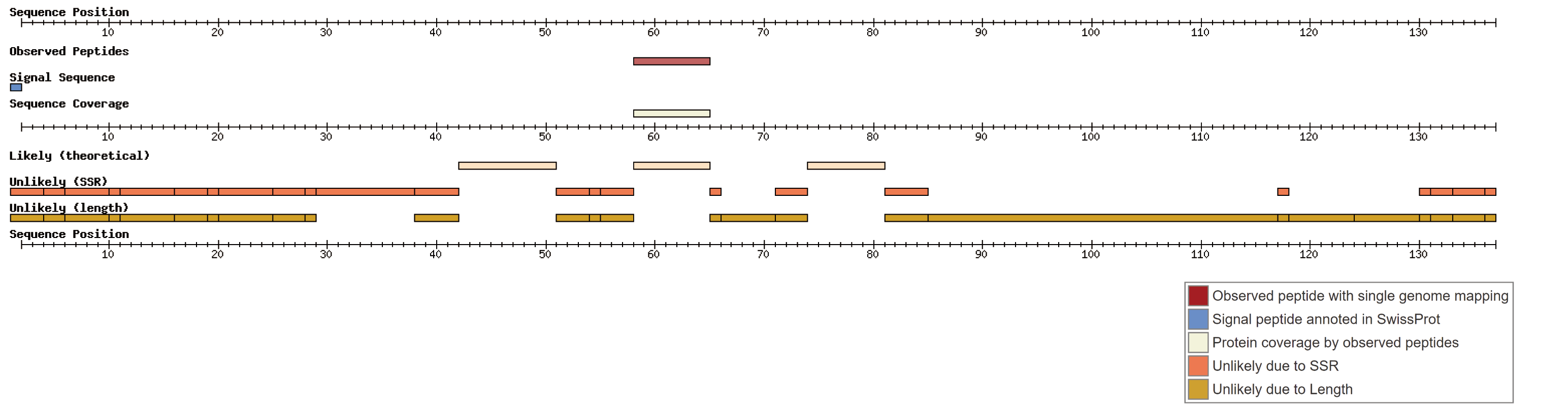

Supplement: Supplementary file 3 — Summary of five categories of the peptides from H3 analyzed via MS analysis. A schematic map from the PeptideAtlas database (http://www.peptideatlas.org/), which is a multi-organism, publicly accessible compendium of peptides identified in a large set of tandem mass spectrometry proteomics experiments, for all possible peptides that could be identified by MS for H3. The amino sequence positions are shown on the top, and the tryptic peptides are shown as colored blocks in the bottom along the sequence position. Different colors indicate different possibility of being identified by MS. There were five categories of the peptides: A, Observed peptide with single genome mapping; B, Signal peptide annotated in SwissProt; C, Protein coverage by observed peptides; D, Unlikely due to SSR; E, Unlikely due to Length. The peptide sequence between the amino acid residues 81 to 137 belongs to the category E, which is unlikely to be identified due to its length. (PNG 88 kb) [file 10815_2021_2314_Fig7_ESM.png]

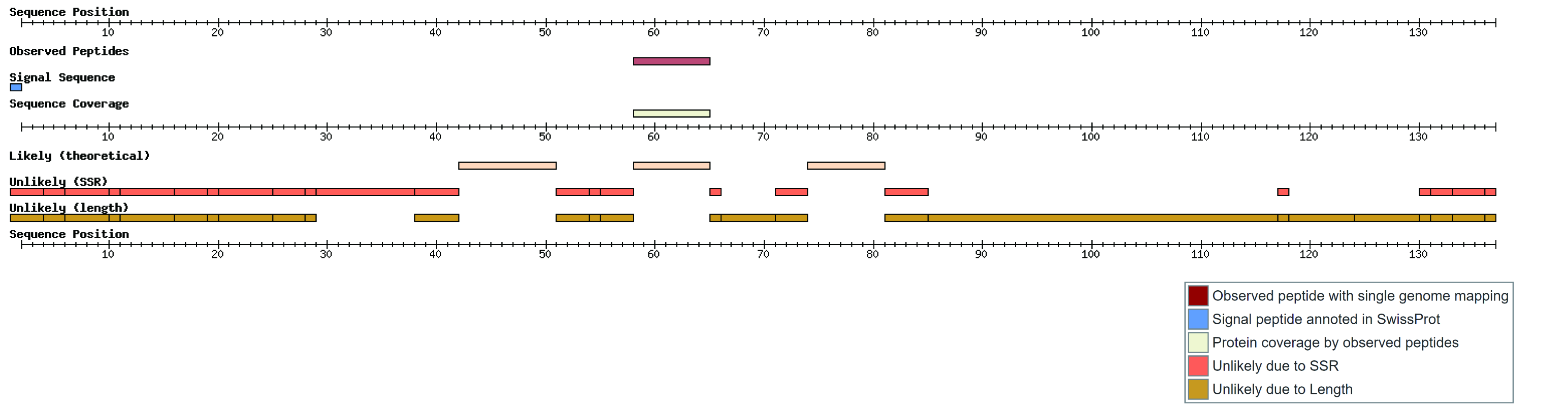

Supplement: Supplementary file 4 — High resolution image (TIF 10281 kb) [file 10815_2021_2314_MOESM2_ESM.tif]
